# The Scandal of Empty Houses

**Published:** 1920-09-11

**Authors:** 


**THE PUBLIC WELL-BEING**—(*continued*).

### The Scandal of Empty Houses.

### AN OVERCROWDED NATION.

A Bill which will have to be closely watched and carefully strengthened in the public interest when it is discussed by the House of Commons on the re-assembly of Parliament is that recently introduced by the Ministry of Health to give power to local authorities to take compulsorily houses suitable for the working classes which have been withheld from occupation for at least three months. It is certainly so much to the good that the Government have now realised the growing scandal of the empty houses. The overcrowding in the slum areas of our great towns is a reproach to public decency. Let any zealous citizen with a comfortable house to live in go down into these districts

**THE PUBLIC WELL-BEING**—*(continued)*

and see for himself the appalling conditions which are the inevitable breeding-grounds of immorality and which must defy, as long as they are allowed to persist, every effort of the public-health organisation to combat or prevent disease. On this very subject Mr. J. H. Thomas delivered a striking speech a few days ago at Portsmouth.

Local authorities who institute prosecutions for overcrowding are beginning at the wrong end. People have to live somewhere; and the obvious remedy is not by way of prosecutions, which can only mean turning them out into the streets, but by finding real homes for them. In Germany the Government decided long ago that all empty houses should be "commandeered" to provide accommodation for homeless people, and no person was allowed more rooms in a house than was sufficient for the needs of his family. Without asking our own Government to go to the length of compelling householders to share their premises with other families, it is surely time that the thousands of houses, large and small, which for various reasons (but chiefly to obtain a high purchase price) are lying empty in all parts of the country should be made available for the public necessities. This would not itself solve the house problem. That is apparent. But it would ameliorate the lot of a large number of unfortunate people and do much towards removing a genuine source of discontent and irritation.

### A Middle-class Plea.

It has been suggested in more than one responsible quarter that the new Bill should be amended by making it compulsory for local authorities to take over all empty houses, without regard to their size or quality, from the date of the passing of the measure, and to offer them to the public at a standard rent. Whatever is done, the prevailing house-hunger is a disease which no Government can afford to disregard indefinitely.

